# High prevalence and emerging positive association of *kelch13* R622I and HRP2-based RDT negativity in *Plasmodium falciparum* in northern Ethiopia

**DOI:** 10.1371/journal.ppat.1013771

**Published:** 2025-12-11

**Authors:** Ayalew Jejaw Zeleke, Abebe A. Fola, George A. Tollefson, Karamako Niaré, Alec Leonetti, Om Taropawala, Jacob Marglous, Rebecca Crudale, Bokretsion G. Brhane, Ashenafi Assefa, Patience Kiyuka, Jonathan B. Parr, Asrat Hailu, Mulugeta Aemero Tegegne, Jeffrey A. Bailey

**Affiliations:** 1 Department of Medical Parasitology, School of Biomedical and Laboratory Sciences, University of Gondar, Gondar, Ethiopia; 2 Department of Pathology and Laboratory Medicine, Brown University, Providence, Rhode Island, United States of America; 3 Center for Computational Molecular Biology, Brown University, Providence, Rhode Island, United States of America; 4 School of Public Health, Brown University, Providence, Rhode Island, United States of America; 5 Ethiopian Public Health Institute, Addis Ababa, Ethiopia; 6 Department of Microbial, Cellular and Molecular Biology, Addis Ababa University, Addis Ababa, Ethiopia; 7 Institute for Global Health and Infectious Diseases, University of North Carolina at Chapel Hill, Chapel Hill, North Carolina, United States of America; 8 Kenya Medical Research Institute, Center for Geographic Medicine Research Coast, Kilifi, Kenya; 9 KEMRI-Wellcome Trust Research Programme, Kilifi, Kenya; 10 Division of Infectious Diseases, Department of Medicine, University of North Carolina at Chapel Hill, Chapel Hill, North Carolina, United States of America; 11 Department of Microbiology, Immunology and Parasitology, Faculty of Medicine, Addis Ababa University, Addis Ababa, Ethiopia; Columbia University Irving Medical Center, UNITED STATES OF AMERICA

## Abstract

The rise of antimalarial drug-resistant *Plasmodium falciparum* threatens malaria elimination efforts. Mutations in the gene *kelch13* (*k13*) confer artemisinin partial resistance (ART-R), compromising the efficacy of frontline artemisinin-based combination therapies (ACTs). The validated mutation *k13* R622I has emerged and expanded rapidly in the Horn of Africa. We conducted a year-long genomic surveillance study in Gondar Zuria and Tach Armachiho, two ecologically distinct districts in northwestern Ethiopia where R622I was first identified. A total of 903 *P. falciparum* infections were sequenced using molecular inversion probe (MIP) panels targeting major drug resistance mutations and genome-wide informative SNPs. The R622I mutation was found in 44.3% of samples, more frequent in Gondar Zuria than Tach Armachiho (52% vs. 35%; *p < 0.001*), and persisted year-round in nearly all sites, indicating stable transmission with minimal seasonal variation. Histidine-rich protein 2 (HRP2) based rapid diagnostic test (RDT) negativity was also prevalent (39.3%), with significant district-level variation (48.7% vs. 27.6%; *p < 0.001*). Concerningly, R622I and HRP2-RDT co-occured in 22% of samples, higher in Gondar Zuria than in Tach Armachiho (28.9% vs. 12.9%; *p < 0.00*1). Overall, HRP2-RDT negativity was significantly more common among R622I mutant parasites than wild-type (48.3% vs. 30.7%; *p* < 0.05). The *k13* C580Y mutation was also detected at very low frequency (0.4%) in Gondar Zuria, representing the first report of this mutation in the Horn of Africa. Long-read whole-genome sequencing showed *k13* flanking haplotypes of C580Y isolates were distinct from Southeast Asian lineages, suggesting a local, *de novo* emergence of African origin. These findings highlight the increasing prevalence and types of ART-R mutations, persistence of *k13* R622I and its increasing association with HRP2-RDT negativity, representing a double threat to malaria control and elimination efforts.

## Author summary

Malaria remains a major public health challenge in sub-Saharan Africa, where control efforts are increasingly threatened by the development of *Plasmodium falciparum* parasite resistance to both drugs and diagnostics. In this study, we analyzed 903 *P. falciparum* cases from two districts in northern Ethiopia and found that nearly half of samples carried the WHO-validated ART-R *k13* R622I mutation. The mutation was detected across all surveyed Kebeles and persisted across seasons, at prevalence exceeding previous reports. Remarkably, we also identified the *k13* C580Y mutation, the most common ART-R marker in Southeast Asia, showing evidence of independent emergence in Africa. Moreover, we observed a high co-occurrence of R622I and HRP2-RDT negativity, suggesting a significant association between R622I mutation and HRP2/3 gene deletions. These two resistance traits, previously observed independently in Ethiopia, now appear to co-occur more frequently than expected by chance, implying a combined survival advantage for parasites resistant to both diagnosis and treatment. This convergence of drug and diagnostic resistance marks an alarming new stage in parasite adaptation in Ethiopia. Although current ACTs remain effective, our findings underscore the urgent need for strengthened genomic surveillance, improved diagnostic tools, and adaptive control strategies to prevent the expansion of these dual-resistant lineages and the newly detected C580Y mutation.

## 1. Introduction

Malaria remains a major public health problem, especially in sub-Saharan Africa. In 2023, the global number of malaria cases and related deaths were 263 million and 597,000, representing a slight increase compared to 2022 [1]. Provision of effective treatment is critical to control malaria and progress towards elimination in addition to effective vector mitigation and control. Artemisinin-based combination therapies (ACTs) are the frontline treatment recommended by the World Health Organization (WHO) for uncomplicated *P. falciparum* [1]. These combination therapies combine a fast-acting artemisinin derivative with a long-acting partner drug in a pairing intended to slow the emergence of drug resistance [2]. In Africa, ACTs were introduced in the early 2000s and have become the cornerstone of treatment across the continent, helping drive the reduction of malaria incidence and mortality [2,3]. Artemether-lumefantrine (AL), Artesunate-amodiaquine (ASAQ), dihydroartemisinin-piperaquine (DP), artesunate-mefloquine (ASMQ), artesunate-sulfadoxine-pyrimethamine (ASSP), and artesunate-pyronaridine (AP) are approved ACTs in Africa. Among these, artemether-lumefantrine (AL) is the most common treatment for uncomplicated falciparum malaria in Africa, accounting for 85% of all ACT purchases [4,5]. Despite combination therapy, artemisinin partial resistance (ART-R) has emerged in Africa, posing a serious threat to malaria control efforts. ART-R is characterized by delayed parasite clearance within the first three days or a parasite clearance half-life (PCT_1/2_) of 5 hours or more after treatment with ACTs. ART-R is attributable to various loss-of-function mutations in the propeller domain of the *kelch13* (*k13*) gene, which were first identified in Western Cambodia between 2006 and 2007 [6,7], and quickly spread to other parts of the Greater Mekong (GM) subregion [8]. This spread was accompanied by the rapid emergence of partner drug resistance, leading to ACT clinical failure in GM subregion [9].

Recent studies have now identified both validated and candidate ART-R mutations in Africa. Since 2014, multiple mutations have been reported across countries including R561H in Rwanda [10], C469Y and A675V in Uganda [11], and R622I in Eritrea [12] and Ethiopia [13]. *k13* P441L has recently emerged but lacks a known singular origin, suddenly appearing at sites in multiple countries: Tanzania [14], Uganda [11], Rwanda [15], Ethiopia [16], Namibia [17] and Zambia [18]. Modeling of longitudinal data from Uganda supports that selection pressure favoring ART-R mutations is acting as strongly in Africa as it did in Southeast Asia (SEA) [19]. All flanking haplotypes for most African mutations are distinct from SEA, consistent with de novo mutation in Africa rather than importation from SEA [14,20]. Other *k13* mutations common in SEA, like C439A and C580Y, have been observed only fleetingly in Africa. It remains unclear whether this absence reflects fundamental biological or selective differences between African and Asian parasite populations, or simply the stochastic emergence of different ART-R mutations across regions.

Three-fourths of Ethiopia’s land mass is malaria endemic, predominating in the west, and with 60% of malaria cases caused by *P. falciparum* [21]. Transmission is highly heterogeneous and seasonal. Malaria has resurged recently, with cases increasing by approximately 154% in 2022 compared to 2021 (from 1.3 to 3.3 million); and further to 4.1 million in 2023. Despite this resurgence, the country is still boldly committed to elimination by 2030. A key component of the elimination program is early diagnosis and treatment with effective medications [22]. AL has been the first-line treatment for uncomplicated falciparum malaria since 2004 in the country [23]. It remains efficacious [24], but detection of the ART-R *k13* R622I mutation [25] and its observed rapid spread have raised concern [13]. Research in the Amhara region of northwest Ethiopia, where R622I was first identified in Africa [25], has shown consistently increasing prevalence over time [16]. Malaria control in the context of emerging drug resistance *k13* mutations is compounded by additional challenges such as seasonal malaria transmission, population mobility, recent conflict, and the growing prevalence of *hrp2/3* gene deletions, which compromise the effectiveness of widely used rapid diagnostic tests [26–28]. To investigate these converging threats, we conducted a genomic surveillance study using samples collected from 903 individuals with *P. falciparum* malaria over a one-year period in two districts of Ethiopia’s Amhara region: Gondar Zuria, a highland area with moderate transmission, and Tach Armachiho, a lowland district with high transmission intensity.

## 2. Results

### 2.1 Study population

**Fig 1:**
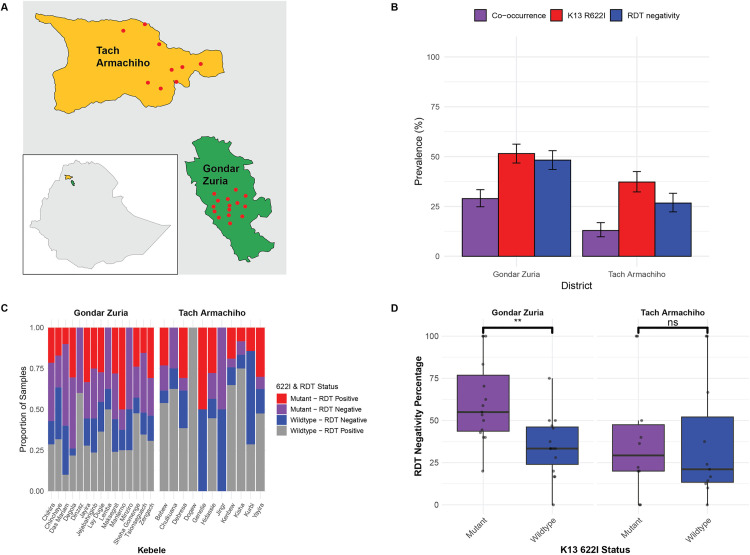
Prevalence of *k13* R622I mutation and RDT negativity in *P. falciparum* infections in Northwest Ethiopia. **(A)** Map of study districts and Keabeles (kebele - red dots) where participants were sampled. Administrative boundary data were obtained from the Global Administrative Areas database (GADM, version 4.1, www.gadm.org). **(B)** Grouped bar plot showing *k13* R622I prevalence and HRP2-based RDT negativity and their co-occurrence rates by district (all enrolled participants had microscopically confirmed *P. falciparum* infection). **(C)** Stacked bar charts showing the distribution of R622I mutation by RDT result by Kebele. **(D)** Prevalence of HRP2-based RDT negativity by *k13* R622I status and district showing significantly higher RDT negativity in mutant infections compared to wildtype infections in Gondar Zuria (Student’s t-test p = 0.0022). No significant difference in RDT negativity percentage was found in Tach Armachiho. Boxplot center lines show the median value, with upper and lower bounds representing the 25th and 75th quartiles, and whiskers extending to 1.5 × IQR from the lower and upper quartiles. Asterisks (*) denote level of statistically significant differences based on Student’s t-test: p < 0.01 (**), and p ≥ 0.05 (ns, not significant).

A total of 903 individuals with microscopy-confirmed *P. falciparum* infections were enrolled in the study. Among them, 55.3% (n = 500) were from Gondar Zuria and 44.6% (n = 403) from Tach Armachiho districts (Fig 1A), encompassing 26 Kebeles (the smallest administrative units in Ethiopia). Overall, 59.4% of participants were male (536/903), with a mean age of 22.1 ± 13.0 years. Fifty-six participants (6.2%) reported having received antimalarial treatment within two weeks prior to sample collection, and 65 (7%) reported recent travel. The median parasite density determined by microscopy was 9,121 parasites/μL (IQR 2,519–22,160) (S1 Fig), and gametocytes were detected in 39 samples (4.4%) (Table 1).

**Table 1:** Socio-demographic and clinical characteristics of study participants.

Variables		Gondar Zuria (n = 500)	Tach Armachiho (n = 403)	Overall (N = 903)
Sex	Male	333(66.6%)	203(50.4%)	536 (59.4%)
	Female	167(33.4%)	200(49.6%)	367 (40.6%)
Age in years	<5	3(0.6%)	7(1.7%)	10 (1.1%)
	5-17	142(28.4)	183(45.4%)	359 (39.8%)
	≥18	355(71.0%)	213(52.9%)	534 (59.1%)
Residence	Rural	382(76.4%)	176(43.7%)	558 (61.8%)
	Urban	118(23.6%)	227(56.3%)	345 (38.2%)
HRP2-based RDT	Positive	230(46.0%)	260(64.5%)	490 (54.3%)
	Negative	218(43.6%)	99(24.6%)	317 (35.1%)
	Not done	52(10.4%)	44(10.9%)	96(10.6%)
Treatment history	Yes	36(7.2%)	20(5.0%)	56 (6.2%)
	No	464(92.8%)	383(95.0%)	847 (92.8%)
Travel history	Yes	44(8.8%)	21(5.2%)	65 (7.2%)
	No	456(91.2%)	382(94.8%)	838 (92.9%)
Gametocyte	Positive	19(3.8%)	20(5.0%)	39 (4.4%)
	Negative	481(96.2%)	383(95.0%)	864 (95.6%)
Successfully sequenced samples for each target gene	*k13*	486(97.2%)	382(94.8%)	868 (96.1%)
	*mdr1*	465(93%)	365(90.6%)	830 (91.9%)
	*crt*	438(87.6%)	361(89.6%)	799(88.5%)
	*dhfr*	451(90.2%)	360(89.3%)	811 (89.8%)
	*dhps*	450(90%)	357(88.6)	807 (89.3%)

Of the 807 participants with HRP2-based RDT results, 39.3% (317/807) tested negative despite microscopy-confirmed *P. falciparum* infection, indicating a substantial false-negative rate and raising concerns about the reliability of HRP2-based RDTs in this region (Table 1). The mean parasite density among RDT-negative cases (geometric mean [GM]=6,388 parasites/μL; 95% CI = 5,172–7,889) was not significantly different from that of RDT-positive cases (GM = 7,862 parasites/μL; 95% CI = 6,778–9,119) (Kruskal–Wallis test, p = 0.1396).

Moreover, 97.3% and 95.9% of the RDT-negative samples had parasitemia levels exceeding 50 and 100 parasites/µL, respectively, which are above the typical detection limit (50 parasites/µL) of SD Bioline HRP2-based RDT used in this study (manufacturer’s specification). This finding suggests that RDT negativity is driven by *hrp2/3* gene deletions rather than low parasite density, consistent with previous reports of high *hrp2/3* deletion frequencies in this area [26,28]. The RDT-negativity rate was significantly higher in Gondar Zuria (48.7%) compared to Tach Armachiho (27.6%) (Pearson χ² = 37.15, p < 0.001) (Fig 1B).

### 2.2 Prevalence and Local Spread of *k13* R622I and HRP2-RDT negativity

Using MIP targeted sequencing; we assessed the entire *k13* gene for prevalence of WHO-validated and candidate mutations as well as any other variations. The validated *k13* R622I mutation was found in 369/833 samples (44.3%, 95% CI 40.9-47.7%) (S2 Fig and S1 Table). For this mutation, 83.7% (309/369) of infections were solely mutant, while the remaining 16.3% (60/369) were mixed infections with wildtype. The prevalence of R622I was significantly (Student’s t-test *p < 0.001*) higher in Gondar Zuria district (highland with lower malaria transmission) (51.5%, 95% CI 46.9-56.0%) compared to Tach Armachiho (lowland with higher malaria transmission) (35.4%, 95% CI 30.5-40.3%) (Fig 1B and S2 Table). The mutation was identified in all but one Kebele (Fig 1C and S3 Table) across both districts suggesting broad spread of this mutation at local scale. There was no significant variation by Kebele (S3 Fig). We also detected C580Y, a previously validated marker in SEA, in two individuals (0.2%). Other variants occurred outside the propeller domain, with only the common polymorphisms K189T (25.2%) and E401Q (3.5%) exceeding 1% frequency (S2 Fig).

Combined R622I-RDT-negative parasites were prevalent in nearly all Kebeles across both districts (Fig 1C). The overall co-occurrence of R622I and RDT negativity was 22.0% (95% CI: 19.0-25.3), with significantly higher prevalence in Gondar Zuria (28.9%, 95% CI: 24.6–33.5) than in Tach Armachiho (12.9%, 95% CI: 9.5–17.0; *p = 0.001*; Fig 1B). Overall, RDT-negativity was significantly higher among parasites with the R622I mutation than wildtypes (48.3% 95% CI 43.0-53.5% vs. 30.7% 95% CI 26.3-35.1%; Student’s t-test *p < 0.05*, S4 Fig). In Gondar Zuria, this RDT negativity rate was significantly higher among mutants (56.2%) compared to wildtypes (39.8%, Student’s t-test *p = 0.002*); in Tach Armachiho, RDT negativity was also higher among mutants than wildtype (34.7% vs 21.9%), but not statistically significant (Fig 1D).

### 2.3 Detection of *k13* C580Y mutation in Ethiopia

**Fig 2:**
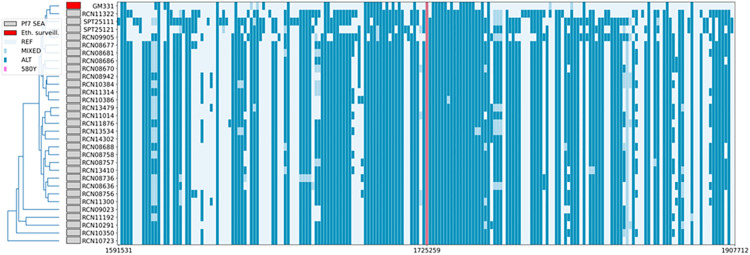
Flanking haplotype comparison of the *k13* locus in Ethiopian C580Y WGS sample compared to SEA *k13* C580Y mutants from Pf7 public database. Dark blue = alternate allele, light blue = reference allele (relative to 3D7) and intermediate blue = mixed genotype.

We detected two cases (0.4%) of the *k13* C580Y mutation, an artemisinin resistance marker that is common in SEA but only occasionally reported in Africa, including Ghana [29] and Nigeria [30]. MIP sequencing confirmed that both samples, from Gondar Zuria district (Jejabahiriginb Kebele), were monogenomic and clonal infections (IBD = 1). Whole-genome sequencing (WGS) using long-read Oxford Nanopore Technology (ONT) following selective whole-genome amplification (sWGA) validated the presence of C580Y mutation in the two samples. We achieved sufficient coverage for flanking haplotype analysis in one sample, with average genome coverage of 51X, and 82% of the genome covered at a depth of at least 5X (S5A Fig). The other sample has validated C580Y but had insufficient coverage (mean coverage 6X, and 36% of the genome had at least 5X coverage) (S5B Fig). The flanking haplotype based on the long-reads from the more deeply sequenced sample was distinct from those in SEA based on comparison to 41 representative publicly-available SEA C580Y parasites [31] (Fig 2). Both C580Y mutant infections were HRP2-based RDT negative, despite high parasitemia levels (28,627 and 12,277 parasites/µL). Further, WGS analysis confirmed the absence of reads mapping to both the *hrp2* and *hrp3* genes in these samples (S5C and S5D Fig). Each *P. falciparum* isolate harboring the *k13* C580Y mutation carried it as a single *k13* variant without additional *k13* mutations. These isolates contained *pfmdr1* Y184F, and the *pfdhfr* triple mutations (N51I, C59R, S108N), while no mutations were detected in *pfdhps* or *pfcrt*.

### 2.4 Prevalence of other key antimalarial drug resistance markers

We analyzed mutations related to partner drug sensitivity, as sustained partner drug efficacy is essential for maintaining the effectiveness of ACTs. In addition to *k13* mutations, we observed near fixation (99.8%) of MDR1 N86 (wildtype), associated with reduced lumefantrine susceptibility [32]. All samples carried D1246 (wildtype) and 96.8% carried 184F (mutant). We found additional novel *mdr1* mutations (e.g., A989E, R627I, D650N, T172K) with low frequency in the population (<1%) (S1 Table). For chloroquine resistance transporter (*crt*) gene, the resistant CVIET haplotype was found in 98.1% of samples, with A220S, N326S, and R371I mutations reported 81.4%, 73.3%, and 92.5% samples, respectively. These *crt* resistance markers were more prevalent in Gondar Zuria than Tach Armachiho (S2 Table). Unlike other East African regions where wild-type *crt* alleles are re-emerging, resistant haplotypes persist in Ethiopia, likely maintained by continued chloroquine use for *P. vivax* [33] (S6A Fig).

As in other African countries, sulfadoxine-pyrimethamine (SP) was withdrawn as a frontline antimalarial in Ethiopia in 2004 and is not used for intermittent preventive treatment during pregnancy (IPTp). Nevertheless, SP resistance mutations remain widespread. In the *P. falciparum* dihydrofolate reductase *(dhfr)* gene, N51I and S108N mutations were detected in 99.4% and 99.2% of samples, respectively, while C59R occurred in 31.1%, and I164L was absent. In the dihydropteroate synthase *(dhps)* gene, A437G and K540E mutations were found in 88.8% and 89.1% of samples, respectively, and A581G in 5.0% (S1 Table). The *dhfr* triple-mutant IRN haplotype was identified in 28.4% of samples, and 86.4% of S108N carriers also harbored the *dhps* K540E mutation (S6B Fig).

### 2.5 Co-occurrence of R622I with *mdr1* and *crt* mutations

**Fig 3:**
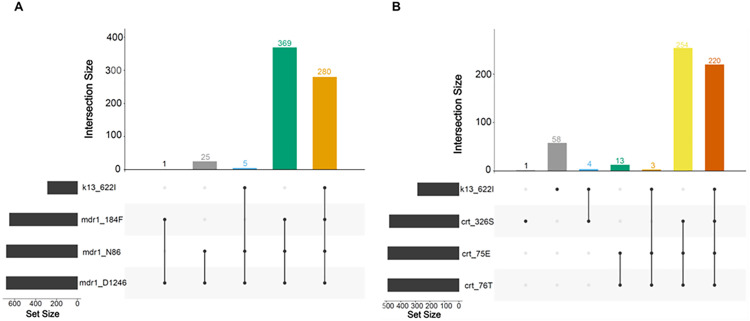
Prevalence of the *k13* R622I mutation and other partner drug-resistance mutations in monoclonal *P. falciparum* infections. **(A)** Combinations of *k13* R622I and *mdr1* drug resistance mutations implicated in lumefantrine susceptibility, including the *mdr1* NFD haplotype (N86 wild type, 184F mutant, and D1246 wild type). **(B)** Combinations of *k13* R622I and *crt* drug resistance mutations implicated in aminoquinoline susceptibility, including *crt* (326S, N75E, and K76T). Only monoclonal samples (n = 680) with genotypes for all loci were included. The “intersection size” on the vertical axis on both panels represents the number of isolates.

Mutations in the *mdr1* gene, including N86 (wild), 184F (mutant), and D1246 (wild), are commonly observed in *P. falciparum* populations. Among monoclonal *k13* R622I parasites, the NFD haplotype (N86/184F/D1246) and the *mdr1* Y184F mutation alone were highly prevalent, detected in 98.2% (95% CI: 96.7–99.8%) of samples (Fig 3A). The wild-type N86 allele was present in all R622I parasites. Moreover, 78.2% (95% CI: 73.5–83.0%) and 78.6% (95% CI: 73.8–83.4%) of R622I monoclonal parasites carried the *crt* K76T and N75E mutations, and the N326S mutation, respectively (Fig 3B). The *crt* K76T mutation was significantly more common among R622I parasites compared to wild types (78.2% vs. 67.6%; Pearson χ² = 9.33, *p* = 0.002). However, linkage disequilibrium (LD) analysis (S7 Fig) revealed weak LD between *crt* and *k13*, suggesting that these mutations likely arose independently rather than through shared selective pressure.

### 2.6 Genetic relatedness and connectivity of *k13* mutant parasites

**Fig 4:**
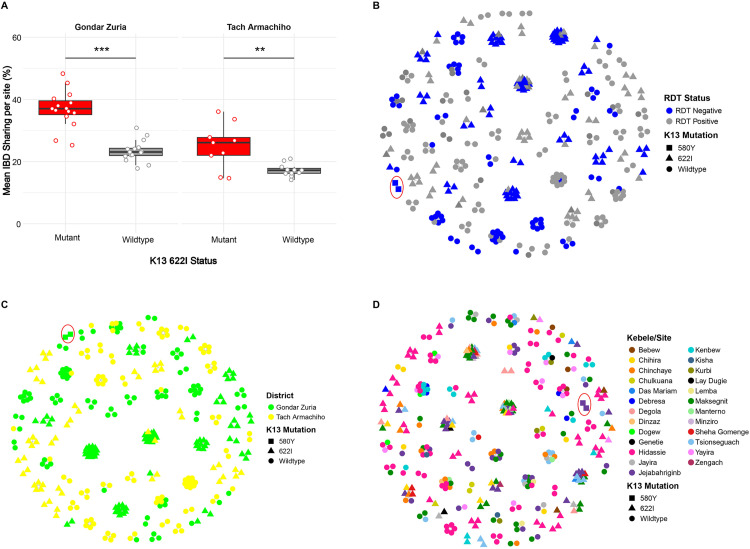
*k13* mutant and wildtype parasite relatedness. **(A)** Pairwise IBD sharing between *k13* R622I mutant and wild-type *P. falciparum* parasites within Kebeles across different study districts. Asterisks (*) denote level of statistically significant differences based on Student’s t-test: p< < 0.01 (**), and p< < 0.001 (***). Boxes indicate the interquartile range; the line indicates the median, the whiskers show the 95% confidence intervals and dots show within-Kebele IBD values within each district. **(B)** Network analysis of highly related parasite pairs (IBD≥ ≥ 0.95) by RDT test results shaped by *k13* mutation status. **(C)** Network analysis of highly related parasite pairs (IBD≥ ≥ 0.95) by district shaped by *k13* mutation status. **(D)** Network analysis of highly related parasites (IBD≥ ≥ 0.95) at Kebele (village) level shaped by *k13* mutation status. Each node identifies a unique isolate, and an edge is drawn between two isolates if they meet the IBD threshold. Within panels B, C, and D; the *k13* C580Y parasites, shaped as rectangles and encircled by red circles are clustered by RDT results, study district and Kebele level, indicating their clonality. Color codes correspond to RDT results, districts and Kebeles.

To assess connectivity of the *k13* R622I mutant parasites population at district and Kebele levels, we analyzed pairwise relatedness using identity by descent (IBD) among monoclonal infections (88% monoclonality was observed in the current study; S8 Fig). In Gondar Zuria, R622I mutant pairs showed significantly higher IBD than WT pairs (0.369 vs 0.234; Student’s t-test p = 3.29e-8). Similarly, in Tach Armachiho, IBD was higher among mutants than wild types (0.249 vs 0.172; Student’s t-test p = 3.55e-3) (Fig 4A). Within the R622I group, RDT-negative infections displayed stronger genetic relatedness (mean IBD = 0.365) than RDT-positive infections (0.284; *p* < 0.001) (S9 Fig). Highly related parasite pairs (IBD ≥ 0.95) formed distinct clusters of R622I mutants grouped by RDT status (Fig 4B).

At a broader scale, *k13* R622I mutants exhibited clear clonal clustering within districts (Fig 4C). At the local level, parasites showed strong clustering within individual Kebeles, with some evidence of genetic connectivity between Kebeles within the same district (Fig 4D). The two *k13* C580Y mutant parasites similarly showed tight clustering by RDT status, district, and Kebele (Fig 4B–4D, outlined in red circle).

### 2.7 Monthly prevalence and clonality of *k13* R622I

**Fig 5:**
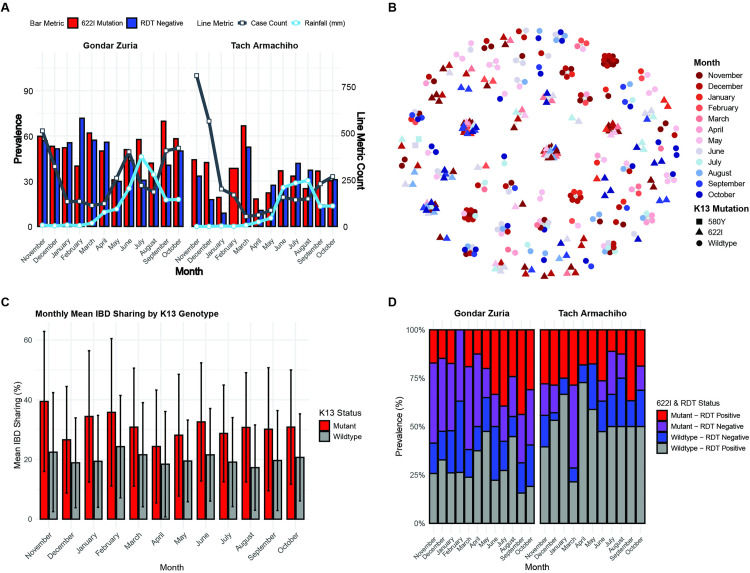
Monthly prevalence of the *k13 R*622I mutation, RDT negativity, *P. falciparum* positivity, and genetic relatedness in parasite samples collected between November 2022 to October 2023 in Gondar Zuria and Tach Armachiho districts, Northwest Ethiopia. **(A)** Monthly prevalence of *k13* R622I mutation and the rate of RDT negativity among *P. falciparum* isolates, along with rainfall and *P. falciparum* case count patterns across months per district. **(B)** Network analysis of highly related monoclonal parasite pairs (IBD ≥ 0.95) by month, where each node represents a unique isolate, and edges are drawn between isolates meeting the IBD threshold; isolates without such connections are not shown, and color codes correspond to different months. Within this panel, C580Y parasites, which are depicted as rectangles and encircled in red, share IBD ≥ 0.95 indicating their clonality and persistence between consecutive months. **(C)** Monthly means pairwise IBD of *P. falciparum* isolates by *k13* genotype status. Error bars represent the 95% confidence intervals. **(D)** Distribution of R622I mutation by RDT result status across months of the study districts.

We next examined the temporal dynamics of the *k13* R622I mutation over the study period (November 1, 2022–October 31, 2023). Although monthly differences were not statistically significant (Mann–Kendall trend test, *p* > 0.05; S10A and S10B Fig), the peak prevalence of R622I reached 60.5% (95% CI: 45.0–76.1%) in March. This peak coincided with the end of the dry season, when rainfall is minimal from November to February and resumes after March. Health center records indicated that *P. falciparum* case numbers were lowest in March, aligning with the R622I prevalence peak. A similar pattern was observed in Tach Armachiho, where prevalence reached 62.5% (95% CI: 38.8–86.2%) in March. In Gondar Zuria, R622I prevalence was slightly higher in September (67.6%, 95% CI: 51.9–83.4%), followed by March (59.1%, 95% CI: 38.5–79.6%). The lowest prevalence occurred in May for both the overall dataset (25.9%, 95% CI: 16.2–35.8%) and Gondar Zuria (27.1%, 95% CI: 15.8–38.5%), while in Tach Armachiho it was lowest in April (18.2%, 95% CI: 4.6–40.9%). Across all months, R622I prevalence remained consistently higher in Gondar Zuria than in Tach Armachiho, except in March (Fig 5A and S4–S6 Tables). These results indicate that the R622I mutation persists year-round, including during periods of low malaria transmission.

IBD network analysis further revealed that R622I mutants were closely related and maintained strong genetic connectivity throughout the study period, whereas wild-type parasites showed greater genetic differentiation over time. R622I mutants from different months formed large interconnected clusters, in contrast to wild-type parasites, which appeared as smaller, temporally restricted clusters (Fig 5B). This pattern is consistent with a higher mean pairwise IBD among mutants compared to wild types across all months (Fig 5C). RDT-negative and R622I mutant parasites were detected every month in Gondar Zuria and in most months in Tach Armachiho. It is also interesting to note that March was the month when the co-occurrence of RDT-negativity and the R622I mutation peaked in both districts (Fig 5D), suggesting RDT negativity may aid in avoiding treatment during low transmission periods.

## 3. Discussion

Through intensive, year-round sampling in two northern Ethiopian districts where the *k13* R622I mutation was first detected in Africa [25], we document a rapid rise and widespread dissemination of this mutation, the predominant artemisinin resistance marker in Horn of Africa. The R622I mutation was detected in nearly half of all infections, reaching 52% prevalence in the highland district of Gondar Zuria. This mutation was detected in nearly all surveyed Kebeles (sites), indicating its local establishment. Compared to wild-type parasites, mutants exhibited high genetic relatedness consistent with more clonal expansion events relative to wildtype [13]. Strikingly, compared to a lack of association previously in 2018 [14], we observed a strong association and co-occurrence between *k13* R622I and HRP2-based RDT negativity, suggesting that parasites simultaneously resistant to both drug and diagnostics may have selective advantage. Such *‘double-resistant parasites’* clonally persists throughout the year, with potential peaks during the dry season, implying that it can endure and spread even during periods of minimal transmission. Additionally, we identified the WHO-validated ART-R *k13* C580Y mutation for the first time in Ethiopia, a previous high-grade ART-R marker in Southeast Asia, and determined it likely originated in Africa. These findings reveal an alarming continued evolution of diagnostic and drug resistance, underscoring the urgent need for enhanced molecular surveillance, improved diagnostic tools, and adaptive control measures..

Our data reveal a steep and alarming rise in the *k13* R622I mutation prevalence in northern western Ethiopia compared to previous years, reaching 44% overall and detected in all surveyed Kebeles. This represents a sharp upward trajectory from earlier low prevalence surveys: 2.4% in 2014 [25], 19.4% in 2021 [34], 46.9% in 2022 [16], and reaching 52% in the lower-transmission district of Gondar Zuria in the present study (S11 Fig), underscoring the success of its spread, likely driven by widespread ACT use. The consistently high prevalence across nearly all sampled sites in both districts, strongly indicates that R622I mutant parasites are now firmly established in northern Ethiopia. R622I mutants exhibited significantly higher mean pairwise IBD values than wild-types [35], consistent with increased selfing and clonal spread [36,37], a pattern mirrored in previous findings [13,16] and observed in neighboring Eritrea [12] where R622I has also risen to high prevalences. The detection of highly related genotypes shared across districts suggests regional connectivity, likely facilitated by seasonal migration from highland to lowland areas for agricultural work between May and November [38]. While modest resistance was measured in Eritrean R622I parasites [12], functional assays in Ethiopia are urgently needed to characterize Ethiopian R622I lineages on delayed parasite clearance and treatment outcomes in Ethiopia.

A key finding of our study is the positive association in parasites of the *k13* R622I mutation and HRP2-based RDT negativity (a proxy for *pfhrp2/3* gene deletions). This observation contrasts with earlier Ethiopian study in 2018 [13] which reported a negative association suggesting emergence in separate lineages. Given the genes responsible for artemisinin resistance (*k13*) and diagnostic evasion (*pfhrp2* gene deletion) are on separate chromosomes, their greater than chance co-occurence in parasites suggests a selective advantage in combination. While numerous drug and diagnostic resistant infections were seen in Eritrea [12], it was unclear if it was simply the high background rate of HRP2 deletions in which *k13* R622I mutation may have arisen or whether they were evolving in preferential co-association. The basis for this synergy is not straight forward. It has been assumed previously that drug and diagnostic resistance would remain anti-correlated because RDT negative parasites would be less likely to encounter drugs thus it increases parasite fitness, obviating the fitness benefit of *k13* mutants that are weaker in the absence of ACTs. We hypothesize the synergy is likely due to the incomplete nature of both resistant mechanisms. First, parasites testing negative by RDT may still be empirically treated with ACTs based on symptoms. Second, delays in treatment due to false-negative RDTs may additionally lead these patients to seek alternative therapies, including low-quality monotherapies. In fact, there is growing evidence that a major driver of ART-R spreads is due to suboptimal drug adherence, a perpetual challenge [39]. Thus, in areas with strong test and treat strategies, drug and diagnostic resistant parasites may thrive the best. Encouragingly, the recent adoption of non-HRP2-based RDTs in Ethiopia and Eritrea may help mitigate this dual selective pressure.

We detected additional validated ART-R *k13* mutations and confirmed an African origin for the C580Y mutation in two samples from the same Kebele. That these two parasites represent a very low frequency (0.4% cases), highlights our study ability to detect what may be emerging in the future as opposed to what is already prevalent. This marker is concerning as it was a major ART *k13* mutation that spread dramatically across SEA [8,40]. While it has been sporadically reported in Africa countries such as in Ghana [29] and Nigeria [30], it has never been confirmed by multiple methods nor the flanking haplotype determined. Our findings, supported by both MIP and whole-genome sequencing, revealed that the two *k13* C580Y positive parasites were monogenomic, highly related (IBD = 1), and carried a distinct haplotype unrelated to SEA lineages, indicating a likely *de novo* local emergence. Notably, these parasites were HRP2-RDT negative and one patient carrying C580Y parasite had recently received artemether–lumefantrine, suggesting possible treatment failure. Thus, despite its rarity, the local emergence of *k13* C580Y underscores the importance of intensified genomic surveillance to better inform control efforts prior to widespread prevalence [41,42].

Beyond *k13* mutations, we observed a high prevalence of *pfcrt* resistance markers, particularly in Gondar Zuria, where higher *P. vivax* co-endemicity may sustain chloroquine use and maintain *pfcrt* selection pressure [43]. Tach Armachiho district, with lower *P. vivax* transmission and more consistent ACT usage, exhibited reduced *pfcrt* prevalence, consistent with prior reports [44]. Interestingly, *pfcrt* mutations were more common in R622I-carrying parasites than in wild-type strains, though linkage disequilibrium between the loci was weak, suggesting independent segregation under convergent ACT-driven selection. Parasites carrying both *pfcrt* and *k13* resistance alleles may experience epistatic interactions that enhance survival under drug pressure. Moreover, the persistence of *pfcrt*-mutant lineages in *P. vivax* co-endemic settings may create a permissive genetic background that facilitates the emergence of the *k13* R622I mutation [45]. Together, these findings point to a complex adaptive landscape in northern Ethiopia, where multiple mechanisms of resistance to treatment and detection are converging to drive parasite evolution.

Temporal trends observed in this study, based on continuous year-round sampling, provide key insights into the epidemiological dynamics of artemisinin-resistant *P. falciparum* in northern Ethiopia. The overall prevalence of the *k13* R622I mutation remains high, possibly reflecting localized shifts in drug pressure due to conflict-related disruptions of malaria control programs [46,47]. Notably, R622I mutant parasites persisted through the dry season, when transmission and vector activity are minimal, indicating their capacity to maintain low-density or chronic infections, reported in other studies [48,49]. Mutation prevalence rose again following the rainy season, coinciding with increased transmission and antimalarial use, suggesting that R622I parasites are well established and capable of year-round circulation and fitness costs observed *in vitro* in terms of growth do not appear to affect the longevity of infections.

ART-R can prolong parasite survival following treatment, increasing the duration during which parasites are exposed solely to the partner drug, thereby elevating the risk of selecting for partner drug resistance [50]. Consistent with this, we confirmed previous reports of wild-type *mdr1* N86 near fixation [44,51,52]. This variant, which reduces lumefantrine susceptibility, likely reflects selection over the chloroquine-resistant *mdr1* N86 mutant following the nationwide shift from chloroquine to artemether–lumefantrine [44,53]. These patterns suggest that *k13* resistance is evolving in response to ongoing ACT pressure within parasite populations shaped by prior chloroquine use. This suggests integrated molecular surveillance will be critical to detect emerging multidrug resistance in Ethiopia.

This study significantly enhances current understanding of *Plasmodium falciparum* resistance dynamics in the Horn of Africa. However, this study has some limitations. The major limitation of this study is the use of RDT negativity, a clinical phenotype, as a surrogate for *pfhrp2/3* gene deletions in microscopy confirmed cases. In this region, where *hrp2/3* deletions are known to be highly prevalent [26,28,54], this is a reasonable assumption, although a small degree of misclassification is possible. The similar distributions of parasitemia by microscopy between RDT-negative and RDT-positive cases, along with almost all RDT-negative samples having parasitemia exceeding the typical detection limit of HRP2-based SD Bioline RDT, strongly support that RDT negativity is a good surrogate of *pfhrp2/3* deletions. However, the absence of molecular typing of *pfhrp2/3* deletions does not present high-resolution analysis of individual *pfhrp2* and *pfhrp3* deletion status. Additionally, data from a single year limit our ability to assess temporal trends in seasonality. Further research on temporal genomics within Ethiopia and nearby countries is warranted.

In conclusion, this study with intensive temporal sampling across two ecologically distinct districts combined with high-resolution targeted sequencing, highlights a growing threat to malaria control, particularly the efficacy of artemisinin-based combination therapies (ACTs) in northern Ethiopia. We report a sharp rise in the prevalence of the *k13* R622I mutation and increased co-occurrence of *k13* R622I with HRP2-based RDT negativity capable of evading both treatment and diagnosis. Crucially, the *k13* C580Y resistance mutation, a key ART-R marker, is reported for the first time in Ethiopia, even if at a low frequency, providing an early warning for control efforts. Collectively, these findings provide critical new insight into *P. falciparum* resistance dynamics in the Horn of Africa and underscore the urgent need for continuous molecular surveillance, improved diagnostic tools, and adaptive intervention strategies to sustain malaria control and elimination efforts.

## 4. Materials and methods

### 4.1 Ethics statement

Ethical clearance was obtained from the University-level Institutional Review Board (Ref: VP/RTT/05/814/2022) and the Institutional Ethics Review Board of the College of Medicine and Health Sciences, University of Gondar (Ref: CMHS/S/H/R/CS/06/214/3/2022). Permissions were also obtained from the respective district Health Offices and Kebele administrations. Formal written informed consent was obtained from all adult participants or from parents or guardians of participating children. In addition, written assent was obtained from children aged 12–17 years. All procedures followed appropriate safety and ethical standards. Screening forms and case records were stored securely with access limited to authorized personnel, and electronic data were coded with unique identifiers. Genotyping work at Brown University was regarded as non-human subject research; only de-identified samples and aggregated clinical data were used for publication.

### 4.2 Study site

The study was conducted from November 01, 2022 to October 31, 2023 in Gondar Zuria and Tach Armachiho districts, Amhara region, northwest Ethiopia (Fig 1A). The Gondar Zuria district is about 45 km away from Gondar town, which is 686 km far from the capital city (Addis Ababa), Ethiopia. The area of Gondar Zuria District is 1108.53 km^2^, with an estimated population size in 2023 of 252,091. It has more than 35 smallest administrative units called Kebeles and is bordered on the south by the Debub Gondar Zone, on the southwest by Lake Tana, on the west by Dembiya, on the north by Lay Armachiho, on the northeast by Wegera, and on the southeast by Mirab Belessa. Its climate is Woina Dega (subtropical zone) with an altitude ranging between 1750 and 2600 meters above sea level. It has an average annual temperature of about 25.1 degree Celsius with annual rainfall between 719.9 and 1831.9 millimeters. During the study period, the district had 8 health centers and 1 hospital. The Tach Armachiho district is located approximately 65 kilometers from Gondar town and 796 kilometers from Addis Ababa. The district is divided into 24 Kebeles and is bordered by Tegedie to the north, Lay Armachiho and Chilga to the south, Metema to the southwest, Mirab Armachiho to the west, and Dabat districts to the east. Its total area is 2,710.41 km^2^ and according to the 2023 estimation, the district’s total population was 121,597. It is a low land area (tropical zone) with an altitude of below 1500 meters above sea level. The mean minimum and maximum temperatures recorded annually are 30°C and 42°C, respectively. The district has a unimodal rainfall pattern and extends from June to the end of September months. Throughout the study period, the district was equipped with 6 health centers and 1 hospital. Over the last of two to three years, there has been a rise in the number of malaria cases reported by the health department of the respective districts. The Ethiopian Federal Ministry of Health’s categorization of malaria transmission intensity places Tach Armachiho district in the high transmission zone, while Gondar Zuria district is identified as having a moderate transmission level.

### 4.3 Sample collection and testing malaria cases

We conducted a health facility-based cross-sectional study over a year. The sources of population were patients who were sent to the laboratory for malaria screening in the selected health centers from the study districts. Maksegnit and Sanja health centers, serving as central hubs for all Kebeles, were chosen from Gondar Zuria and Tach Armachiho districts, respectively. Microscopically confirmed *P. falciparum* infected patients, ≥ 2 years of age & signed informed written consent were included in the study as study population. Patients who were not volunteers, have bleeding disorder and with severe malaria symptoms were excluded from the study. All *P. falciparum* positive patients (n = 903, GZ = 500, TA = 403) who fulfilled the inclusion criteria within the study period were included in the study. Finger prick blood samples (~375 µL) were collected using EDTA-coated microtainer tubes.

Thin and thick smears were made for confirmation and quantification of parasite densities by expert microscopists. Both the thick and thin blood films were prepared from finger prick blood on a single slide and stained using Giemsa solution as described before. Parasitic stage densities were determined using previously used protocol [55]. An SD Bioline rapid diagnostic test (product code 05FK60, Standard Diagnostics, Suwon, South Korea) targeting *P. falciparum* histidine-rich protein 2 (HRP2) was also done. RDTs are chromatographic tests for in vitro diagnosis of malaria parasites. They contain a membrane strip pre-coated with a monoclonal antibody against malaria parasite antigens as a single line across the test strip conjugated to a signal, typically colloidal gold [55]. The monoclonal antibodies were species specific. The RDTs were performed according to the manufacturer’s instructions. Its detection limit was 50 parasites/µL for the HRP2 antigen (manufacturer’s note). Data for all patients screened for malaria from November 1, 2022, to October 31, 2023, at the two health centers were extracted from laboratory log books. Rainfall data were obtained from the CHIRPS: Rainfall Estimates from Rain Gauge and Satellite Observations v3.0 dataset [56] using the longitude and latitude coordinates of each sampled Kebele. The average rainfall values for each sampled Kebele from each district were used to represent the rainfall pattern of the study districts.

### 4.4 DNA extraction and MIP sequencing

Dried blood spots (DBS) were prepared on filter papers (Whatman, Maidstone, UK) for molecular assays as previously described [16]. In brief, the DBS samples were air dried and kept in zip-locked plastic bags containing self-indicating silica gel desiccant beads (Sigma-Aldrich) and transported to University of Gondar and stored at -20^o^C freezer until they were transported to Brown University, Rhode Island, USA. DNA was extracted from DBS (one full 6mm DBS spot, typically involving 2 or 3 punches) using Chelex-Tween as previously described [57].

Five microliters of extracted DNA were used for each of the MIP captures using two panels: one covering key *P. falciparum* drug resistance genes and mutations (DR23KE), including *k13*, *mdr1, crt, dhfr,* and *dhps* genes, and a newly designed panel of 2128 MIPs (IBC2FULL) targeting common SNPs (>5%) across *P. falciparum* genome [58]. MIP capture and library preparation were performed as previously described [13,59]. Sequencing was conducted using an Illumina NextSeq 550 instrument (150 bp paired-end reads) at Brown University (RI, USA). For samples with newly detected *k13* C580Y mutation in Ethiopia, an additional MIP capture was done using the same MIP panel and re-sequenced to a high depth to confirm the mutations. Controls for each MIP capture and sequencing included genomic DNA from serial dilution of lab strain 3D7 as well as no template and no probe controls.

### 4.5 MIP data analysis

Processing of sequencing data and variant calling was done using MIPtools (v0.19.12.13; https://github.com/bailey-lab/MIPTools), a suite of computational tools designed to process sequencing data from MIPs. Raw reads from each MIP, identifiable using unique molecular identifiers (UMIs), were used to reconstruct sequences using MIP Wrangler, and variant calling was performed on these samples using Freebayes [60]. Variants were annotated using the 3D7 v3 reference genome. For the genome-wide MIP panel, variants were quality filtered by removing those with less than 3 UMIs within a sample and less than 10 UMIs across the entire population. To reduce false positives due to PCR and alignment errors, the alternative allele (SNP) must have been supported by more than one UMI within a sample, and the allele must have been represented by at least 10 UMIs across the entire population. Biallelic, variant SNP positions were retained for downstream analyses. Moreover, SNPs with more than 50% missing data, followed by samples with less than 50% data, were removed from all downstream analyses.

### 4.6 Analysis of drug resistance prevalence

The panel for detecting drug resistance included key and known SNP in genes such as *k13, crt, dhfr, dhps, mdr1*, along with other candidate drug resistance genes, as previously mentioned [61]. The rate of mutations associated with drug resistance was determined by dividing the number of infections carrying these mutations by the total number of successfully sequenced infections, then multiplying by 100 to get the prevalence. This filtering process of variants and estimation of drug resistance mutations utilized the miplicorn R package version 0.2.90 (https://github.com/bailey-lab/miplicorn), and the vcfR R package version 1.13.0 from (https://github.com/daniellyer/vcfR). Graphs displaying combinations of drug-resistant mutations were generated and displayed using the ‘UpSetR’ Package in R version 1.4.0 from R Project Repository [62]. Testing for district-level temporal trends in *k13* 622I prevalence and RDT-negativity rate was performed using the “Kendall” package in R version 2.2.1 from the R Project Repository.

We calculated pairwise linkage disequilibrium (LD) between *crt* and *k13* variants in monoclonal infections using custom R scripts. Biallelic SNPs were extracted from the VCF and encoded as binary genotypes. For each variant pair, we computed LD statistics (D, D′, r²) based on haplotype frequencies, where D measures deviation from independence, D′ is the standardized association, and r² is the squared correlation between alleles. Resulting r² values were visualized as a heatmap using ggplot2 to assess inter-locus associations.

### 4.7 Complexity of infection and parasite relatedness analysis

Using the final variant set (n = 2472 SNPs) spread across the genome, we calculated the complexity of infection (COI) for each sample utilizing THE REAL McCOIL categorical method [63]. This method converts heterozygous SNP data into reliable estimates of allele frequencies, achieved through Markov chain Monte Carlo (MCMC) methods. We employed the hmmIBD method to estimate the genetic relatedness among parasite strains samples [64]. Samples were considered to be identical-by-descent if they shared IBD ≥ 0.95 based on a minimum of 50 informative SNPs. We used likelihood-ratio test statistics to test the null hypothesis that two samples are unrelated (H0: IBD = 0) at significance level α = 0.05 (with the procedure adjusted for a one-sided test). We assessed mean IBD and the number of related samples for different groups and performed permutation tests to determine which combinations had higher mean IBD and more related samples than expected by chance. Relatedness networks of parasites were created using the R igraph package.

### 4.8 Long-read whole-genome sequencing and haplotype analysis

Whole-genome sequencing using Oxford Nanopore Technology (ONT) P2 Solo was done on 2 DNA samples (samples with *k13* C580Y mutations based MIP sequencing) to confirm the presence of the mutation within kelch13 propeller domain region of *k13* gene and to assess whether the mutation is locally emerged or imported using extended haplotypes analysis. To get enough template for nanopore sequencing, samples were subjected to selective whole-genome amplification (sWGA) with minor modifications. In brief sWGA selectively amplifies the target genome (here *Plasmodium falciparum* DNA) over background DNA (human genome) using a pool of primers designed to amplify frequently occurring motifs of short nucleotides in *P. falciparum* reference genome. The sWGA experiment was performed in two steps. Combining: 8 ul DNA sample, 0.25 µL (20uM concentration of each primer in the pool), 0.5 µL of 10X ThermoFisher EquiPhi29 reaction buffer, and 1.25 µL nuclease-free water, the resulting 10uL was denatured for 3 minutes at 95º. 2^nd^ The denatured product was then mixed with 1 ul (10 units) of EquiPhi29 DNA polymerase, 2 µL reaction buffer 0.2 µL of 100µM MDT, 2µL of 10mM dNTPs, and nuclease-free water to make a total pool volume of 20µL and isothermally amplified 45ºC for 3 hours, then 65ºC for 10 minutes to suspend further enzyme activity. Amplification success was validated with measuring DNA quantity before and after enrichment using Qubit. The sWGA product was then prepared for sequencing on the Nanopore P2 Solo using the Native Barcoding Kit V14 (SQK-NBD114.24) and the Ligation Sequencing gDNA protocol.

For comparison of C580Y haplotypes, publically-available whole-genome sequencing from SEA with C580Y mutation (n = 41) [31]. High-accuracy duplex base-calling model was done by using Dorado to increase accuracy and alignment was done with minimap tools followed by clair3 variant calling [65]. The *k13* flanking haplotypes for C580Y Ethiopian and SEA strains were visualized by merging variant calls at shared sites and applying a custom plotting script. Although coverage was poor for one of the two long-read sequenced Ethiopian C580Y mutant parasites, the other sample had sufficient coverage and was used for phylogenetic analysis. This well-covered sample was confirmed to have a distinct flanking haplotype in the region surrounding the mutation of interest.

## Supporting information

S1 Fig Histogram showing the distribution of *P. falciparum* parasite density (measured by microscopy) in samples from Northwest Ethiopia. The median density was 9,121 parasites/μL (red line), with the 25th and 75th percentiles at 2,519 and 22,160 parasites/μL, respectively (green lines). One outlier sample with 495,686 parasites/μL was excluded to enhance clarity of the distribution.
(TIF)

S2 Fig Prevalence of *k13* gene mutations in samples collected between November 1, 2022, and October 31, 2023, from Gondar Zuria and Tach Armachiho districts in Northwest Ethiopia. (TIF)

S3 Fig Prevalence of the *k13* R622I mutation across sampled Kebeles within each study district. Error bars represent the 95% confidence intervals of the prevalence estimates.
(TIF)

S4 Fig Prevalence of HRP2-based RDT negativity stratified by *k13* R622I mutation status. The boxplot displays the median (center line), with the box edges indicating the 25th and 75th percentiles.Student’s *t*-test: *, *p* < 0.01.
(TIF)

S5 Fig Genome coverage and evidence of *hrp2/3* gene deletions in C580Y mutant parasites based on whole-genome sequencing. (**A**) Genome coverage of C580Y mutant from sample GM331, with 82% of the genome covered at ≥5 × depth. (**B**) Genome coverage of C580Y mutant from sample GM363, with 36% of the genome covered at ≥5 × depth. (**C**) The BAM file coverage plot shows evidence of *hrp2* gene deletion in the C580Y mutant, indicated by the broken red rectangular regions. (**D**) The BAM file coverage plot shows evidence of *hrp3* gene deletion in the same parasite, indicated by the broken red rectangular regions. The white spaces C and D represent genomic regions with a complete absence of sequencing reads, consistent with gene deletion.
(TIF)

S6 Fig Prevalence of mutations in key antimalarial drug resistance genes. (**A**) Combinations of *crt* gene mutations associated with drug resistance. (**B**) Combinations of *dhps* and *dhfr* gene mutations linked to antifolate resistance.
(TIF)

S7 Fig Linkage disequilibrium (LD) heatmap of *P. falciparum* drug-resistance–associated mutations. The heatmap shows pairwise LD between single nucleotide polymorphisms (SNPs) across *crt*, *dhfr*, *dhps*, *k13*, and *mdr1*. The color scale indicates the strength and direction of LD (red = positive association, blue = negative association, white = no association). NA values along the diagonal represent self-comparisons. Asterisks denote the level of statistical significance (*p* < 0.05, *p < 0.01, **p < 0.001). Clusters of strong LD are evident among *crt* mutations and among the classical *dhfr* triple mutant loci, whereas *k13-Arg622Ile* shows no strong association with *crt* variants.
(TIF)

S8 Fig Frequency of samples by complexity of infection (COI) category, with nearly 88% classified as monoclonal infections. (TIF)

S9 Fig Mean identity-by-descent (IBD) sharing among *k13* R622I parasites, stratified by *HRP2*-based RDT result. IBD sharing was higher among RDT-negative samples (Student’s *t*-test; **** (*p* < 0.0001).
(TIF)

S10 Fig Monthly distribution of *k13* R622I prevalence in two study districts. Panel **A** shows data from Tach Armachiho, and Panel **B** from Gondar Zuria. Error bars represent 95% confidence intervals. Error bars represent the 95% confidence intervals.
(TIF)

S11 Fig Increasing trend in prevalence of the *k13* R622I mutation in *P. falciparum* isolates in Ethiopia from 2014 to 2023, based on previous studies and the present finding. (TIF)

S1 Table Overall prevalence of key mutation among successfully genotyped drug resistance markers, N = number of samples successfully sequenced, n = number of samples with mutation and, % = prevalence of mutation. (XLSX)

S2 Table Prevalence of key mutation among successfully genotyped drug resistance markers from each study district, n = number of samples successfully sequenced, and % = prevalence of mutation. (XLSX)

S3 Table Prevalence of key mutation per Kebeles, n = number samples sequenced, and % = prevalence of key mutation. (XLSX)

S4 Table Overall prevalence of *k13* R622I mutation per month, n = number of carriers for mutation, N = denominator (number of samples successfully sequenced), and % = 622I prevalence. (XLSX)

S5 Table Monthly prevalence of *k13* R622I mutation in Gondar Zuria district, n = number of carriers for mutation, N = denominator (number of samples successfully sequenced), and % = R622I prevalence. (XLSX)

S6 Table Monthly prevalence of *k13* R622I mutation in Tach Armachiho district, n = number of carriers for mutation, N = denominator (number of samples successfully sequenced), and % = R622I Prevalence. (XLSX)

S1 Data Metadata and genotype file for all successfully sequenced samples for drug resistance MIP panel. Only successfully sequenced samples were used for drug resistance marker prevalence estimates. 0 = Reference Call, 1 = Alternative heterozygous call, 2 = Alternative homozygous call, -1 = Missing call. Note that the number of samples successfully genotyped varies per locus drug resistance MIP panel.
(XLSX)
