# Supplementary figures and images for: High prevalence and emerging positive association of kelch13 R622I and HRP2-based RDT negativity in Plasmodium falciparum in northern Ethiopia
Source: PLoS Pathog. 2025 Dec 11;21(12):e1013771. doi: 10.1371/journal.ppat.1013771 (PMC12697994; doi:10.1371/journal.ppat.1013771)

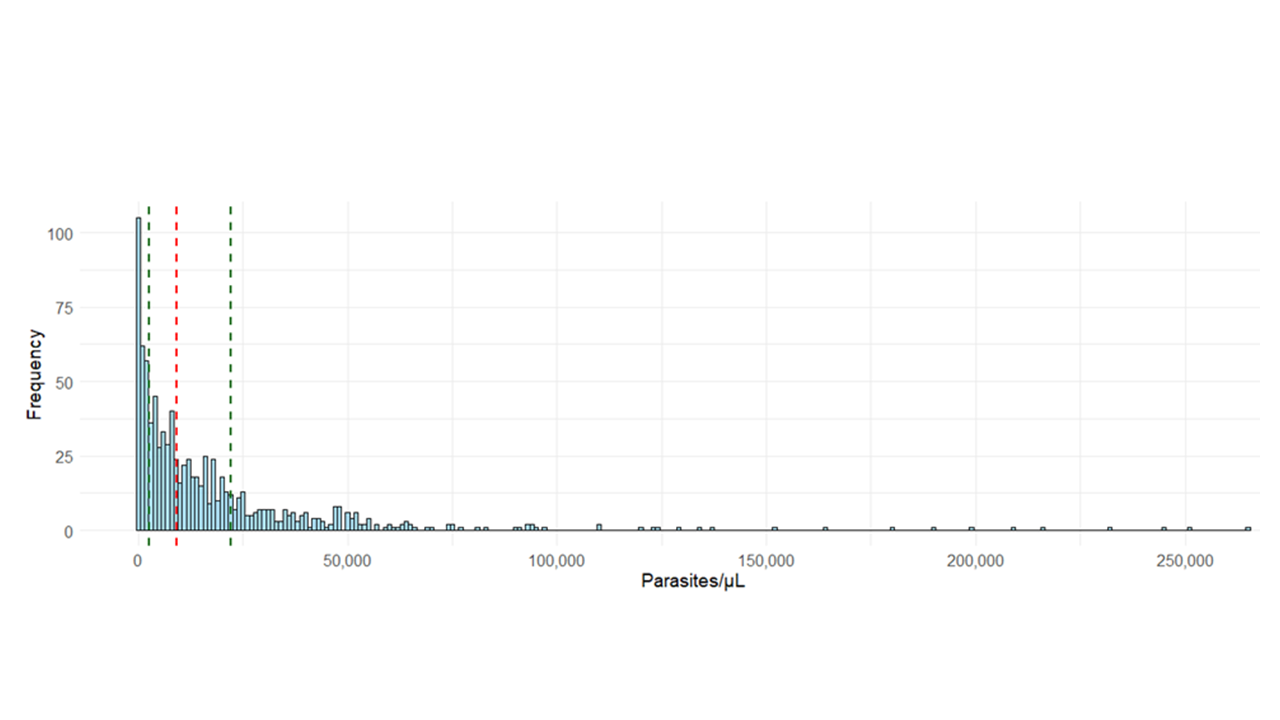

Supplement: S1 Fig — The median density was 9,121 parasites/μL (red line), with the 25th and 75th percentiles at 2,519 and 22,160 parasites/μL, respectively (green lines). One outlier sample with 495,686 parasites/μL was excluded to enhance clarity of the distribution. (TIF) [file ppat.1013771.s001.tif]

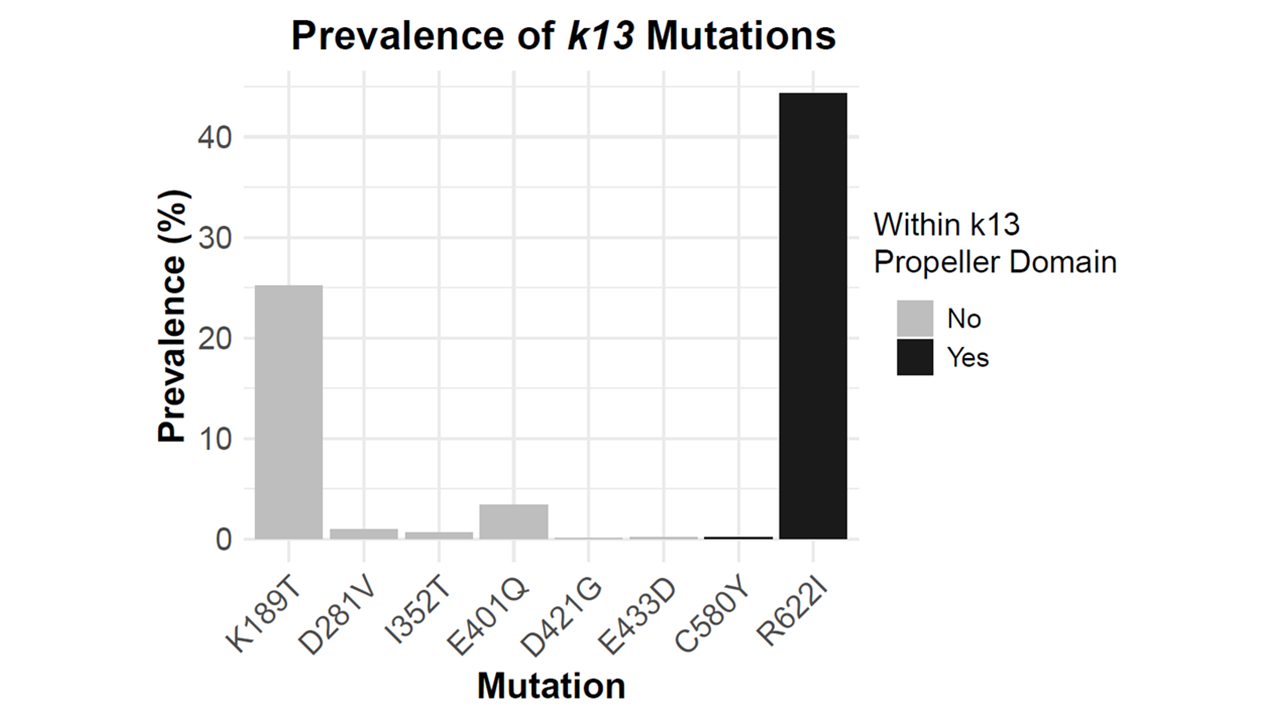

Supplement: S2 Fig — (TIF) [file ppat.1013771.s002.tif]

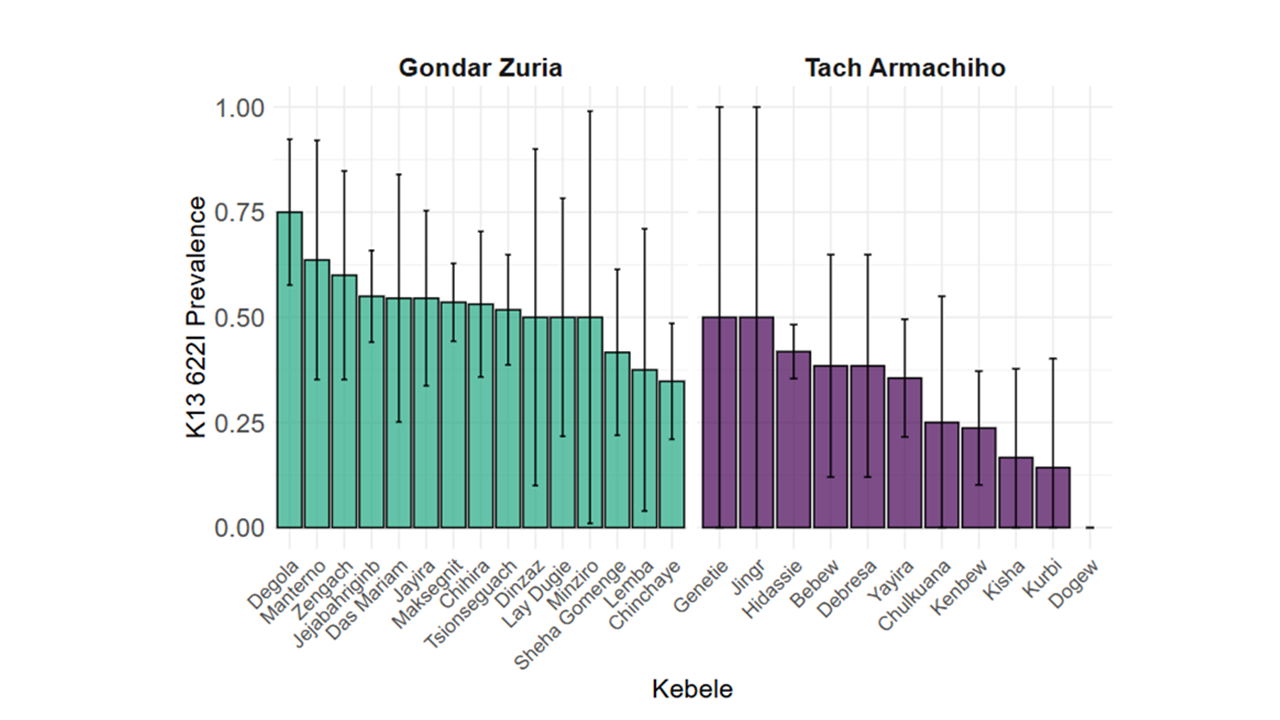

Supplement: S3 Fig — Error bars represent the 95% confidence intervals of the prevalence estimates. (TIF) [file ppat.1013771.s003.tif]

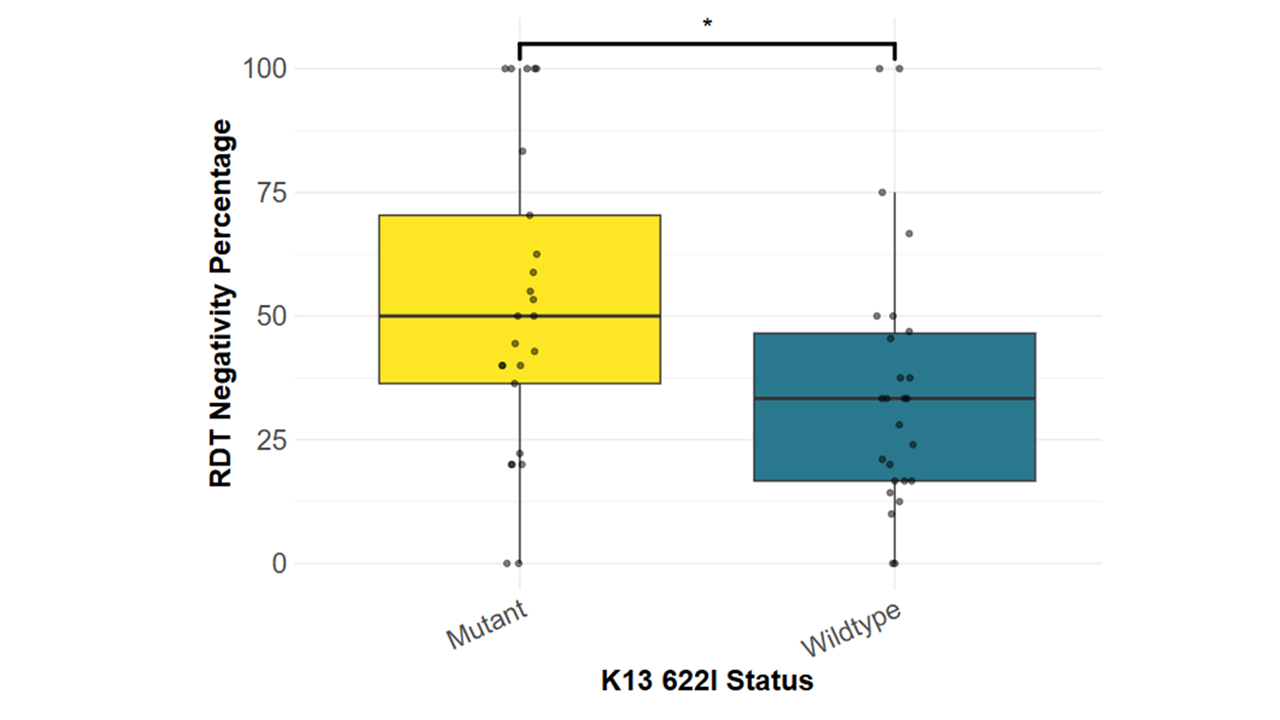

Supplement: S4 Fig — The boxplot displays the median (center line), with the box edges indicating the 25th and 75th percentiles.Student’s t-test: *, p < 0.01. (TIF) [file ppat.1013771.s004.tif]

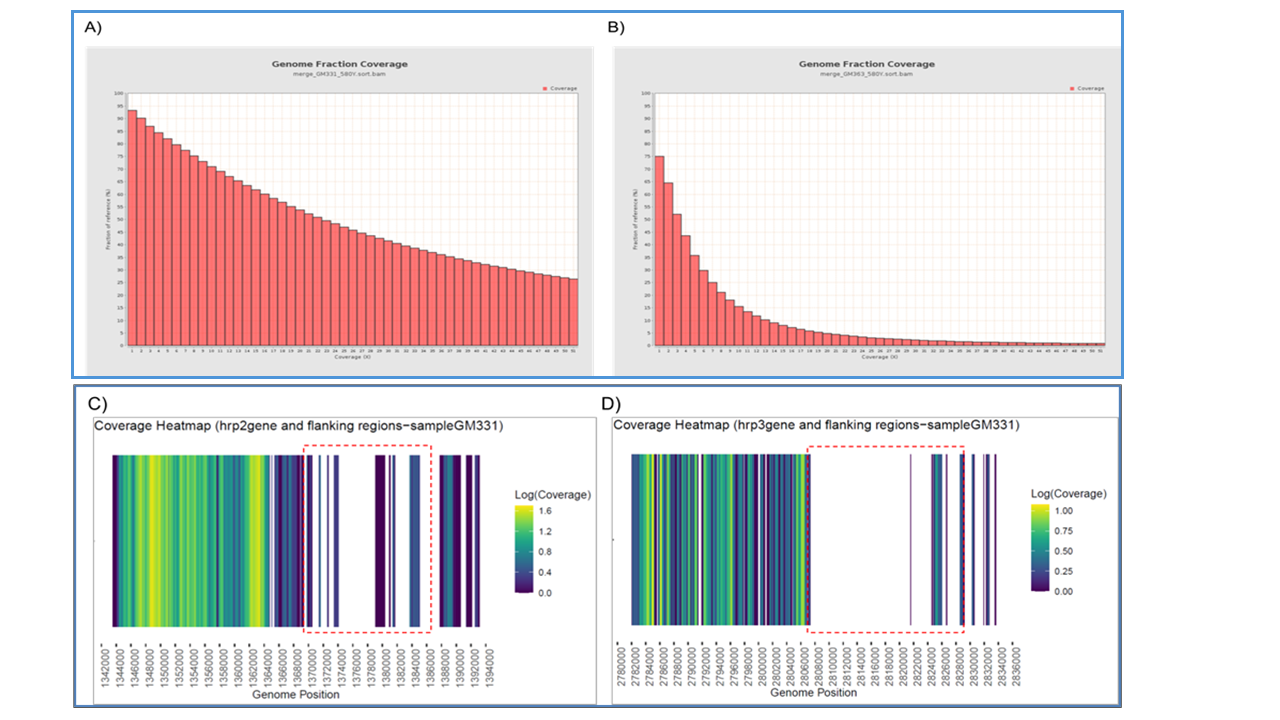

Supplement: S5 Fig — (A) Genome coverage of C580Y mutant from sample GM331, with 82% of the genome covered at ≥5 × depth. (B) Genome coverage of C580Y mutant from sample GM363, with 36% of the genome covered at ≥5 × depth. (C) The BAM file coverage plot shows evidence of hrp2 gene deletion in the C580Y mutant, indicated by the broken red rectangular regions. (D) The BAM file coverage plot shows evidence of hrp3 gene deletion in the same parasite, indicated by the broken red rectangular regions. The white spaces C and D represent genomic regions with a complete absence of sequencing reads, consistent with gene deletion. (TIF) [file ppat.1013771.s005.tif]

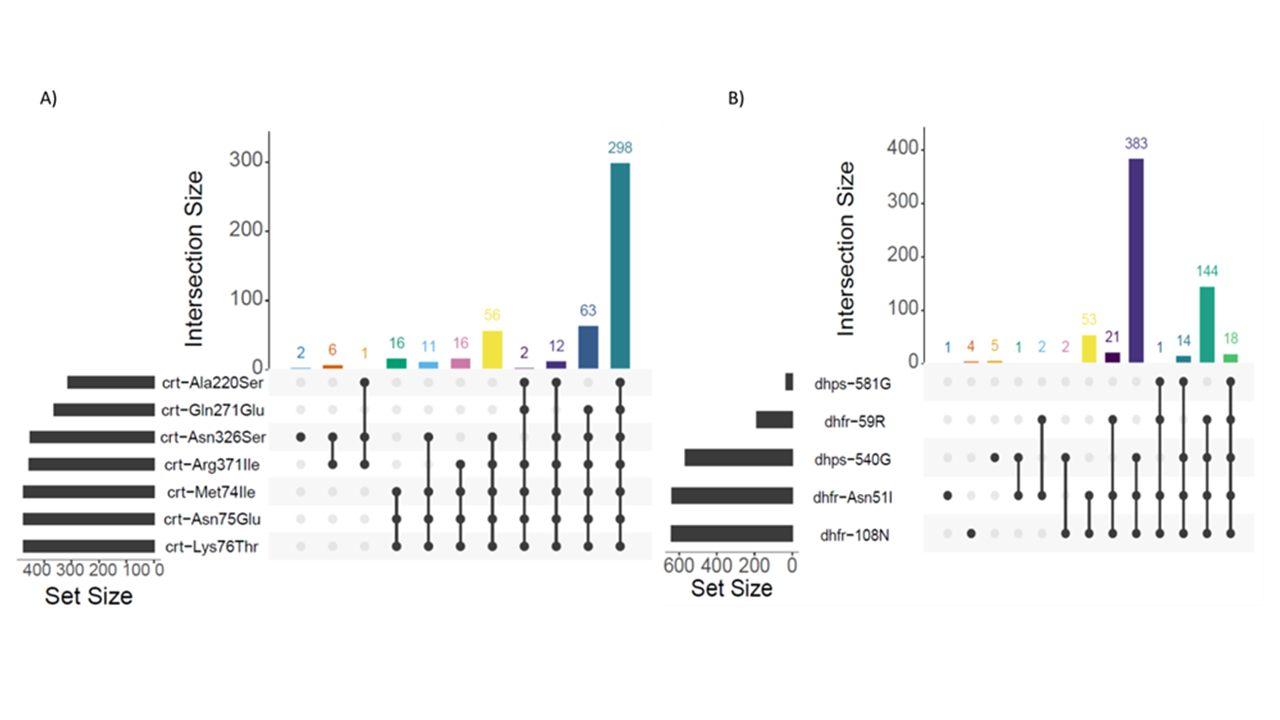

Supplement: S6 Fig — (A) Combinations of crt gene mutations associated with drug resistance. (B) Combinations of dhps and dhfr gene mutations linked to antifolate resistance. (TIF) [file ppat.1013771.s006.tif]

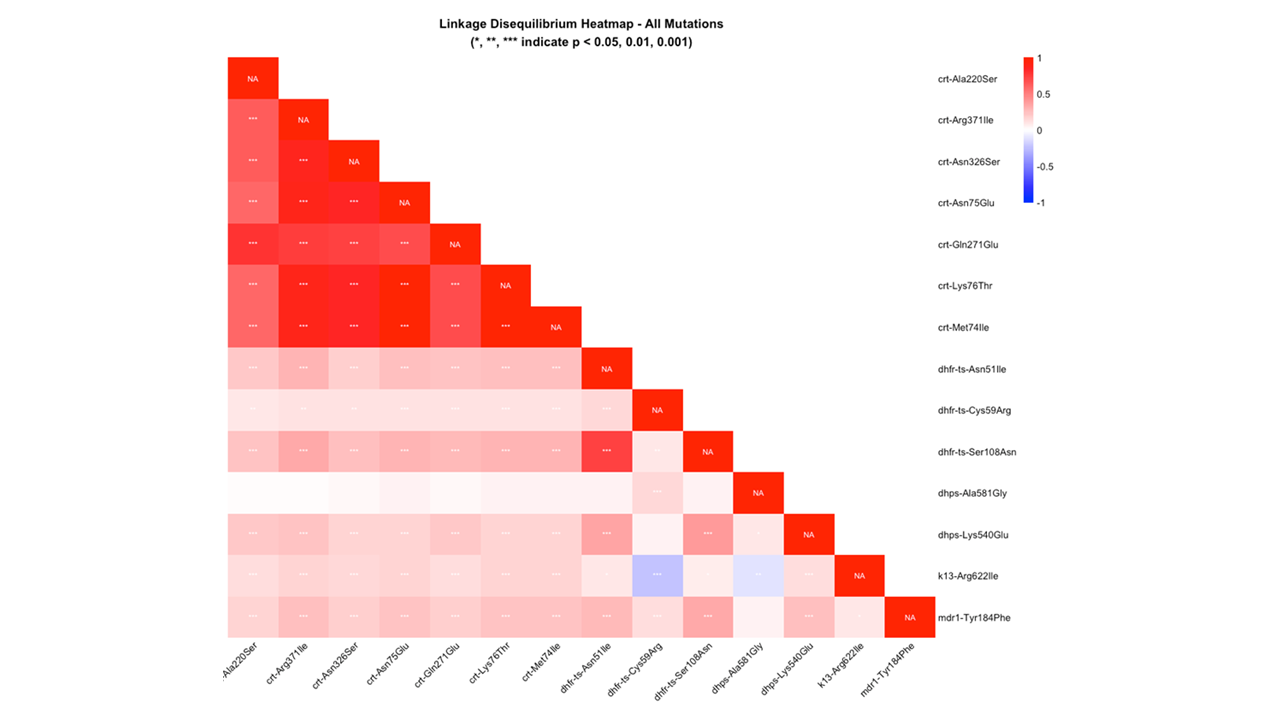

Supplement: S7 Fig — The heatmap shows pairwise LD between single nucleotide polymorphisms (SNPs) across crt, dhfr, dhps, k13, and mdr1. The color scale indicates the strength and direction of LD (red = positive association, blue = negative association, white = no association). NA values along the diagonal represent self-comparisons. Asterisks denote the level of statistical significance (p < 0.05, *p < 0.01, **p < 0.001). Clusters of strong LD are evident among crt mutations and among the classical dhfr triple mutant loci, whereas k13-Arg622Ile shows no strong association with crt variants. (TIF) [file ppat.1013771.s007.tif]

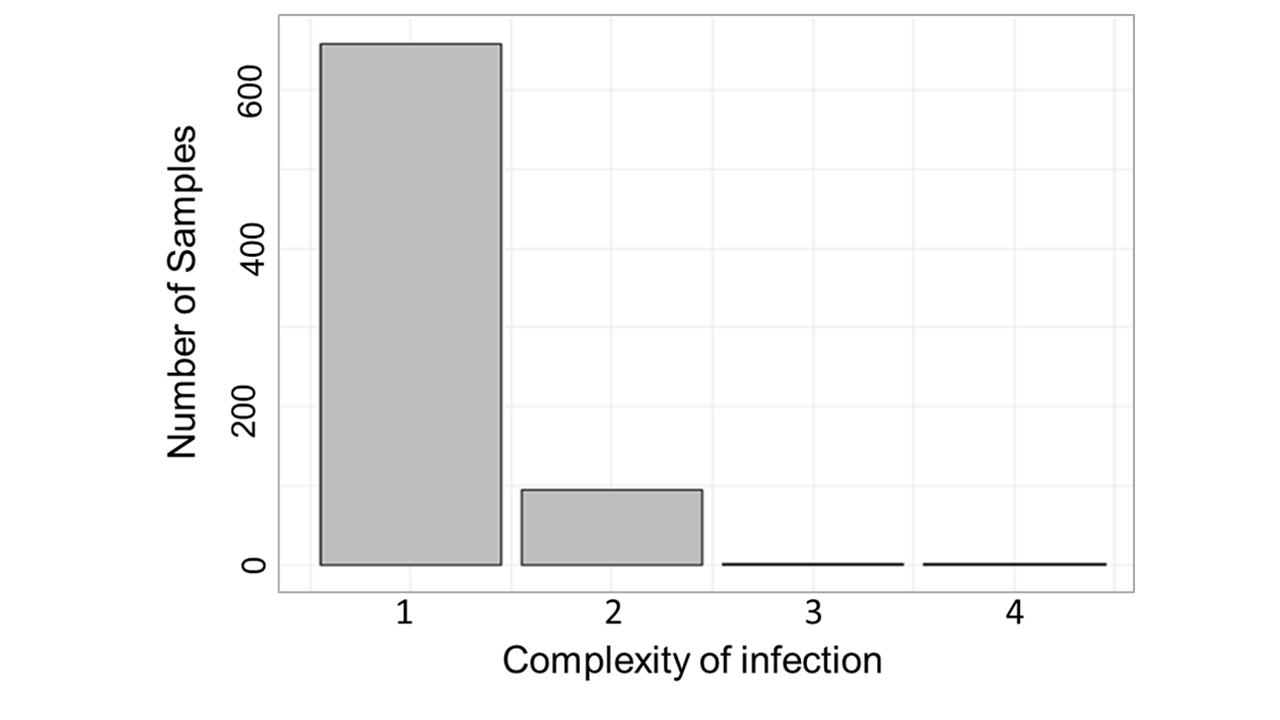

Supplement: S8 Fig — (TIF) [file ppat.1013771.s008.tif]

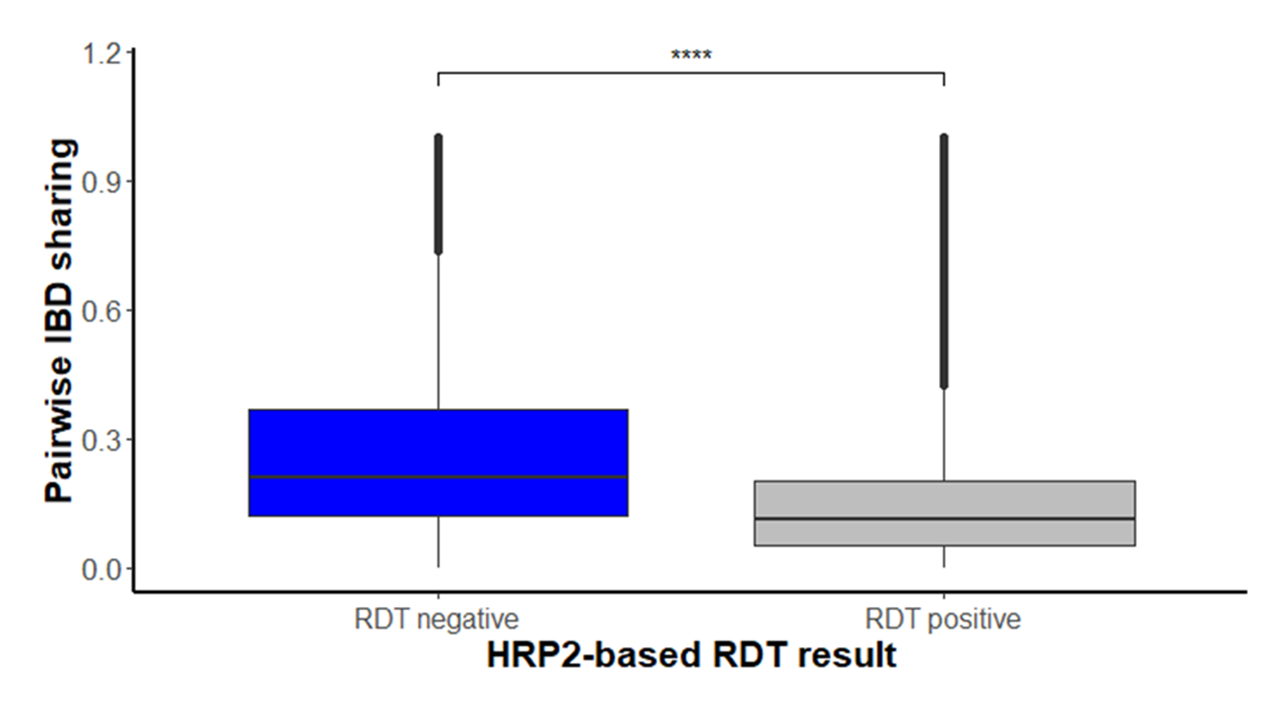

Supplement: S9 Fig — IBD sharing was higher among RDT-negative samples (Student’s t-test; **** (p < 0.0001). (TIF) [file ppat.1013771.s009.tif]

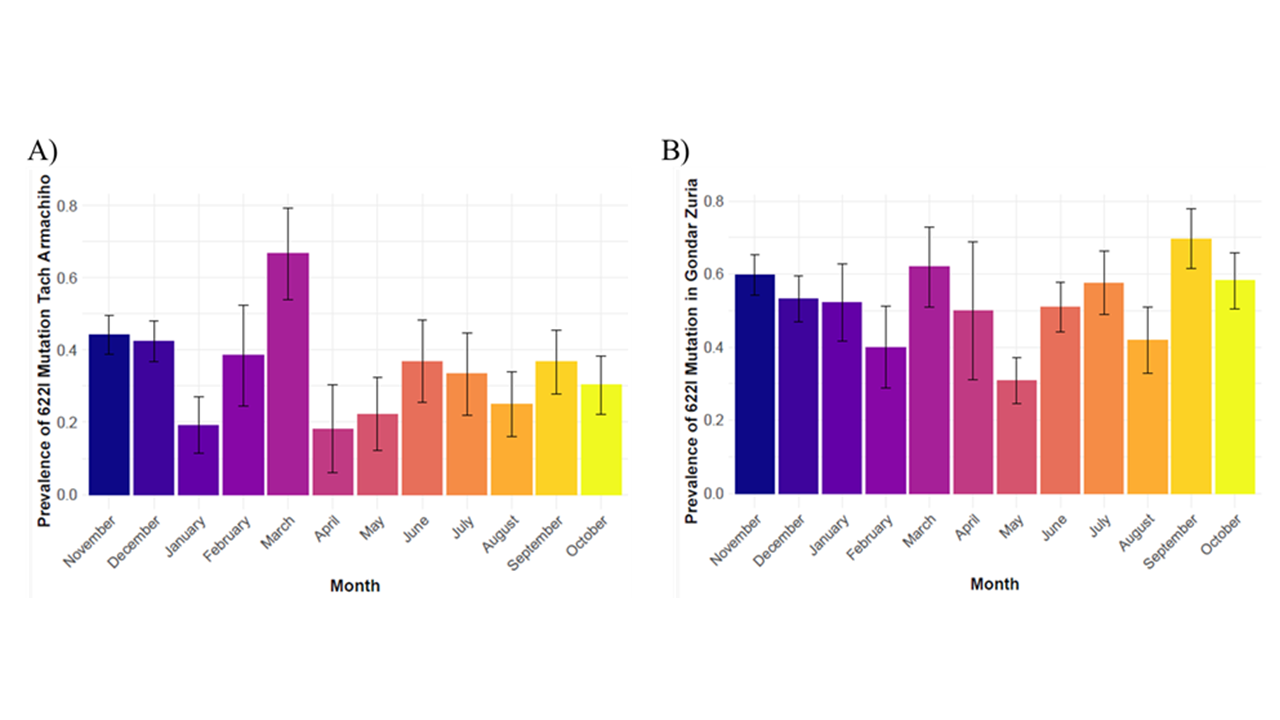

Supplement: S10 Fig — Panel A shows data from Tach Armachiho, and Panel B from Gondar Zuria. Error bars represent 95% confidence intervals. Error bars represent the 95% confidence intervals. (TIF) [file ppat.1013771.s010.tif]

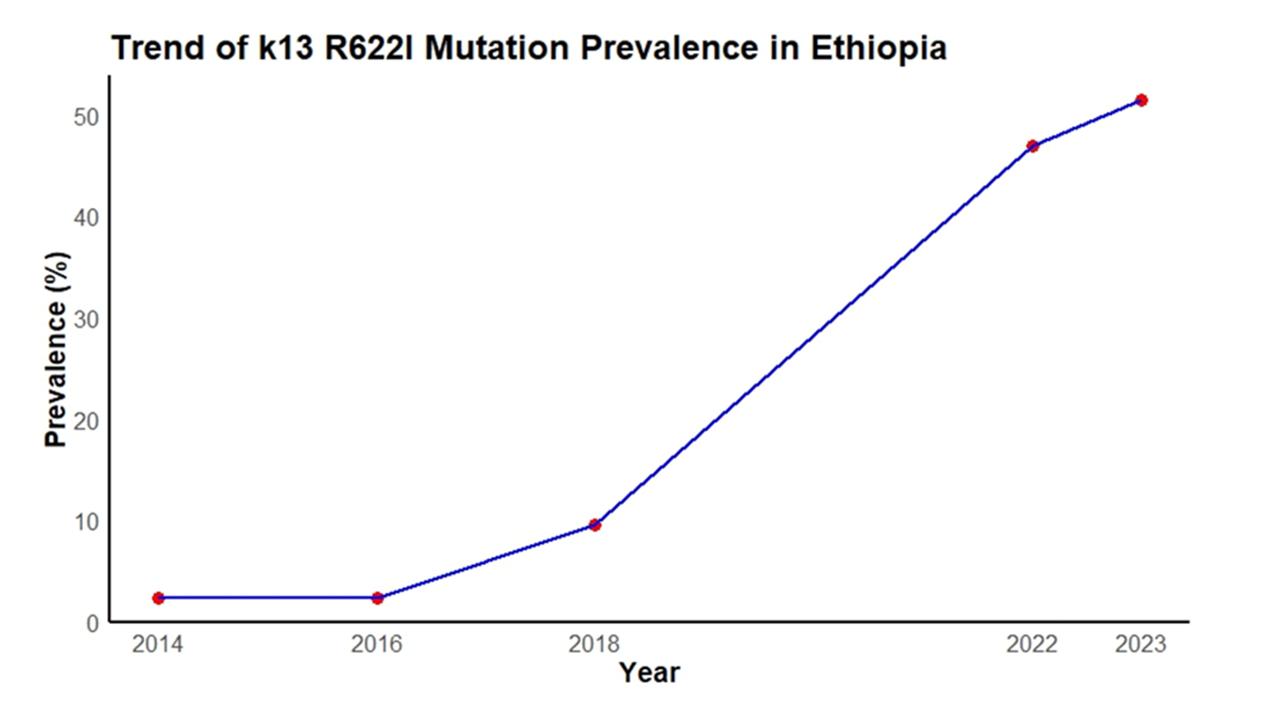

Supplement: S11 Fig — (TIF) [file ppat.1013771.s011.tif]
